# Personalized at-home neurofeedback compared with long-acting methylphenidate in an european non-inferiority randomized trial in children with ADHD

**DOI:** 10.1186/s12888-019-2218-0

**Published:** 2019-08-01

**Authors:** Stéphanie Bioulac, Diane Purper-Ouakil, Tomas Ros, Hilario Blasco-Fontecilla, Marie Prats, Louis Mayaud, Daniel Brandeis

**Affiliations:** 1 CHU Pellegrin, Clinique du Sommeil, F-33076 Bordeaux, France; 2 Université de Bordeaux, Sommeil, Addiction et Neuropsychiatrie, USR 3413, F-33000 Bordeaux, France; 3 CNRS, SANPSY, USR 3413, F-33000 Bordeaux, France; 4 Unit of Child and Adolescent Psychiatry (MPEA1), CHU Montpellier-Saint Eloi Hospital, Montpellier, France; 5 Department of Neurosciences, Laboratory for Neurology and Imaging of Cognition, University of Geneva, Geneva, Switzerland; 6 Department of Psychiatry, Segovia de Arana Health Research Institute (IDIPHISA)-Puerta de Hierro University Hospital, Avenida Manuel de Falla s/n, Majadahonda, Madrid, Spain; 7 Autonoma University, Madrid, Spain; 8 Mensia Technologies, 130, rue de Lourmel, 75015 Paris, France; 9 University of Zurich and ETH Zurich, Neuroscience Center Zurich, Zurich, Switzerland; 10 Department of Child and Adolescent Psychiatry and Psychotherapy, Central Institute of Mental Health, Medical Faculty Mannheim/Heidelberg University, Mannheim, Germany

**Keywords:** Attention deficit hyperactivity disorder, Neurofeedback, Methylphenidate, Clinical trial

## Abstract

**Background:**

Neurofeedback (NF) has gained increasing interest among non-pharmacological treatments for Attention Deficit Hyperactivity Disorder (ADHD). NF training aims to enhance self-regulation of brain activities. The goal of the NEWROFEED study is to assess the efficacy of a new personalized NF training device, using two different protocols according to each child’s electroencephalographic pattern, and designed for use at home. This study is a non-inferiority trial comparing NF to methylphenidate.

**Methods:**

The study is a prospective, multicentre, randomized, reference drug-controlled trial. One hundred seventy-nine children with ADHD, aged 7 to 13 years will be recruited in 13 clinical centres from 5 European countries. Subjects will be randomized to two groups: NF group (Neurofeedback Training Group) and MPH group (Methylphenidate group). Outcome measures include clinicians, parents and teachers’ assessments, attention measures and quantitative EEG (qEEG). Patients undergo eight visits over a three-month period: pre-inclusion visit, inclusion visit, 4 “discovery” (NF group) or titration visits (MPH group), an intermediate and a final visit. Patients will be randomized to either the MPH or NF group. Children in the NF group will undergo either an SMR or a Theta/Beta training protocol according to their baselineTheta/Beta Ratio obtained from the qEEG.

**Discussion:**

This is the first non-inferiority study between a personalized NF device and pharmacological treatment. Innovative aspects of Mensia Koala™ include the personalization of the training protocol according to initial qEEG characteristics (SMR or Theta/Beta training protocols) and an improved accessibility of NF due to the opportunity to train at home with monitoring by the clinician through a dedicated web portal.

**Trial registration:**

NCT02778360. Date registration (retrospectively registered)**:** 5-12-2016. Registered May 19, 2016

## Background

Attention deficit hyperactivity disorder (ADHD) is one of the most common childhood and adolescent psychiatric disorders with prevalence rates ranging from 5 to 7% worldwide [1, 2]. It is a developmental disorder characterized by difficulties in attention, hyperactivity and impulsivity with significant impairment across youth’s social, cognitive, academic, behavioral, and familial functioning [3].

Multimodal approaches are recommended for the treatment of ADHD, consisting of combination of pharmacological and psychological treatments [4]. Pharmacological treatments are efficacious, and form the most frequent and convenient treatment for ADHD in developed countries [5]. However, their benefits may be limited in various situations because of adverse effects, poor adherence or negative medication-related attitudes from parents and clinicians [5, 6]. Thus, it is important to consider non-pharmacological treatments such as psychological and remediation-based strategies. Among non-pharmacological interventions, neurofeedback (NF) has been considered a promising ADHD treatment strategy [6–10].

NF is a computer-based behaviour training enabling a person to self-regulate aspects of brain activity [11, 12], which are commonly measured with electroencephalography (EEG). The incoming EEG signal is compared to a predetermined training goal and this information is then presented to the subject in real-time and reinforced with visual or auditory feedback [13]. The achievement of the target parameter is associated with rewards according to the principles of operant conditioning or skill learning. In practical terms, this takes the form of a computer game in which children obtain rewards when their brain activity changes in the desired direction. Repeated training is thought to foster automatization of the learned process with subsequent changes at the behavioural level [14].

The following training protocols are considered standard NF procedures in ADHD [15]: 1) Theta/beta ratio (TBR) down-regulation, 2) increase in Sensori-Motor Rythm (SMR) and 3) training of Slow Cortical Potentials (SCP). The TBR down-regulation protocol involves reducing theta activity (4–8 Hz) while increasing beta activity (16–20 Hz). It is based on increased slow frequency oscillations (theta) and decreased high frequency oscillations (beta) in subjects with ADHD [16]. However, more recent studies are not in favor of consistent theta or theta/beta increases in ADHD [17] or suggest they may affect a subgroup of 25–40% of patients [18].

The increase in SMR, a low beta frequency observed over the sensory-motor cortex (SMR; 12–15 Hz) has been related to improved hyperactivity and distractibility as well as to a better sleep quality [19, 20]. NF training of slow cortical potentials (SCP) is aimed at learning bidirectional regulation of cortical excitability [21, 22]. The rationale for SCP training is the reduced contingent negative variation (CNV) in ADHD, an event-related potential component associated with cognitive anticipation [23].

NF efficacy in ADHD is a hotly-debated subject [6, 8, 10, 24, 25]. Although several meta-analyses of NF report satisfactory and good clinical effects when based on parents’ assessments [26], other studies have reported no significant effects when “probably blinded ratings” were considered [8, 27]. A network meta-analysis showed superiority of stimulants combined with behavioral therapy or with non-stimulants in comparison with neurofeedback on a dichotomous outcome of ADHD core-symptoms or global functioning [28].There were no differences of acceptability in this meta-analysis between NF and stimulants. However, comparative evidence was likely to be limited by the combined analysis of both methylphenidate (MPH) and amphetamines and by the use of a categorical and global outcome. A more recent meta-analysis included treatment effects at follow-up. Within-group NF effects were of medium effect size on inattention at post-treatment with increasing ES at follow-up and of medium effect size on hyperactivity/impulsivity at both endpoint and follow-up. Medication showed large ES for inattention and medium ES for hyperactivity/impulsivity at both endpoints [29].

Analysis of NF effectiveness is also affected by the diversity of training procedures and methodological issues. In this regard, novel aspects of the NEWROFEED study include a personalized selection of training protocols to match individual EEG characteristics (e.g. based on the individual theta/beta ratio), and improved access through parent-managed training at home coupled with consistent monitoring.

The aim of the NEWROFEED study is to assess the efficacy of a new personalized neurofeedback training device, Mensia Koala™, designed for individualized use at home, through a non-inferiority trial comparing EEG-NF to methylphenidate in children with ADHD.

## Methods

The study is a prospective, multicentre, randomized, reference drug-controlled trial in 179 ADHD children. Subjects are randomized in two groups: NFT group (Neurofeedback Training Group) and MPH group (Methylphenidate group). The study is drug reference-controlled in a 3/2 ratio that maximizes exposure to NFT without impacting power

### Objectives

The main objective of this study is to demonstrate the non-inferiority of a personalized Neurofeedback Training device (i.e. Mensia Koala™) versus Methylphenidate (Medikinet®) in the treatment of children with ADHD. The primary endpoint is the change from baseline (inclusion visit) to end of treatment (last visit) in the clinician-rated ADHD-RS IV total score [30].

The secondary objectives are to investigate whether there are effects on:

- Clinician ADHD RS IV Inattention and Hyperactivity Scores
- Clinical responders (defined as patients reducing their symptoms by more than 25% from their baseline [31]) vs. non-responders
- Parent’s ADHD RS IV Total, Inattention and Hyperactivity Scores
- Teacher’s ADHD RS IV Total, Inattention and Hyperactivity Scores, and Strengths and Difficulties Questionnaire (SDQ)
- Executive Function as measured by the Behavior Rating Inventory of Executive Function (BRIEF)
- Clinical Global Impression (CGI) scale (both components: Severity, CGI-S, and Improvement, CGI-I)
- The Conners Continuous Performance Test 3rd Edition (Conners CPT 3) [32]
- EEG signature evaluation and normalization including individual alpha peak frequency, Quantitative EEG (qEEG) outcome and learning biomarkers.

We hypothesized that the decrease in the Clinician ADHD RS IV total score between baseline (at the inclusion visit) and end of treatment (at final visit) is not superior (i.e. not larger) in the MPH group compared to the NF group.

#### Subjects

The study population includes children diagnosed with inattentive or combined presentation of ADHD. One hundred seventy-nine children aged between 7 to 13 years will be recruited. Clinical diagnosis of ADHD is made by a psychiatrist using Kiddie-SADS (K-SADS) [33], a semi-structured interview with the child and his/her parents.

Thirteen clinical centres from 5 European countries (Belgium, France, Germany, Spain, and Switzerland) will participate in the study.

### Eligibility criteria

A subject is eligible for inclusion in this study only if all of the following criteria apply:

- Children or adolescents (male or female) aged 7–13 years
- ADHD diagnosis positive with K-SADS
- Patient having already had non-pharmacological treatment or psychoeducation for ADHD
- Signature of informed consent form by parent and child
- Wireless internet connection at home

A subject is not eligible for inclusion in this study if any of the following criteria apply:

- ADHD with hyperactive/impulsive presentation without inattention component
- Established diagnostic of epilepsy, autism, schizophrenia or other neurological disorders
- Major psychiatric disorder other than ADHD diagnosed with Kiddie-Sads such as autism, schizophrenia, severe generalized anxiety disorder, major depression, or tics
- Patient having already been treated with psycho-active drug (MPH and others) or EEG-NF for ADHD
- Unable to use the NF device (tablet use and/or headset set-up and/or understanding instructions) according to the investigator
- Medical disorder requiring systemic chronic medication with confounding psychoactive effects
- IQ < 80 using the 3 subtest form of the WASI or the WISC
- Plans to move requiring school change during the next 6 months
- Plans to start other ADHD treatment, including psychotherapy, cognitive behaviour training in the next 6 months
- Patient with chronic medical illness, such as seizure, cardiac disorders, untreated thyroid disease, glaucoma
- Significant suicidal risk based on clinical opinion

### Study flow

Patients undergo eight visits over three months: pre-inclusion visit, inclusion visit (D0), four discovery (NFT group) or titration visits (MPH group), an intermediate visit (D60) and a final visit (D90). Patients are randomized to either the MPH or NFT group at inclusion visit and treated until final visit.

**Fig. 1:**
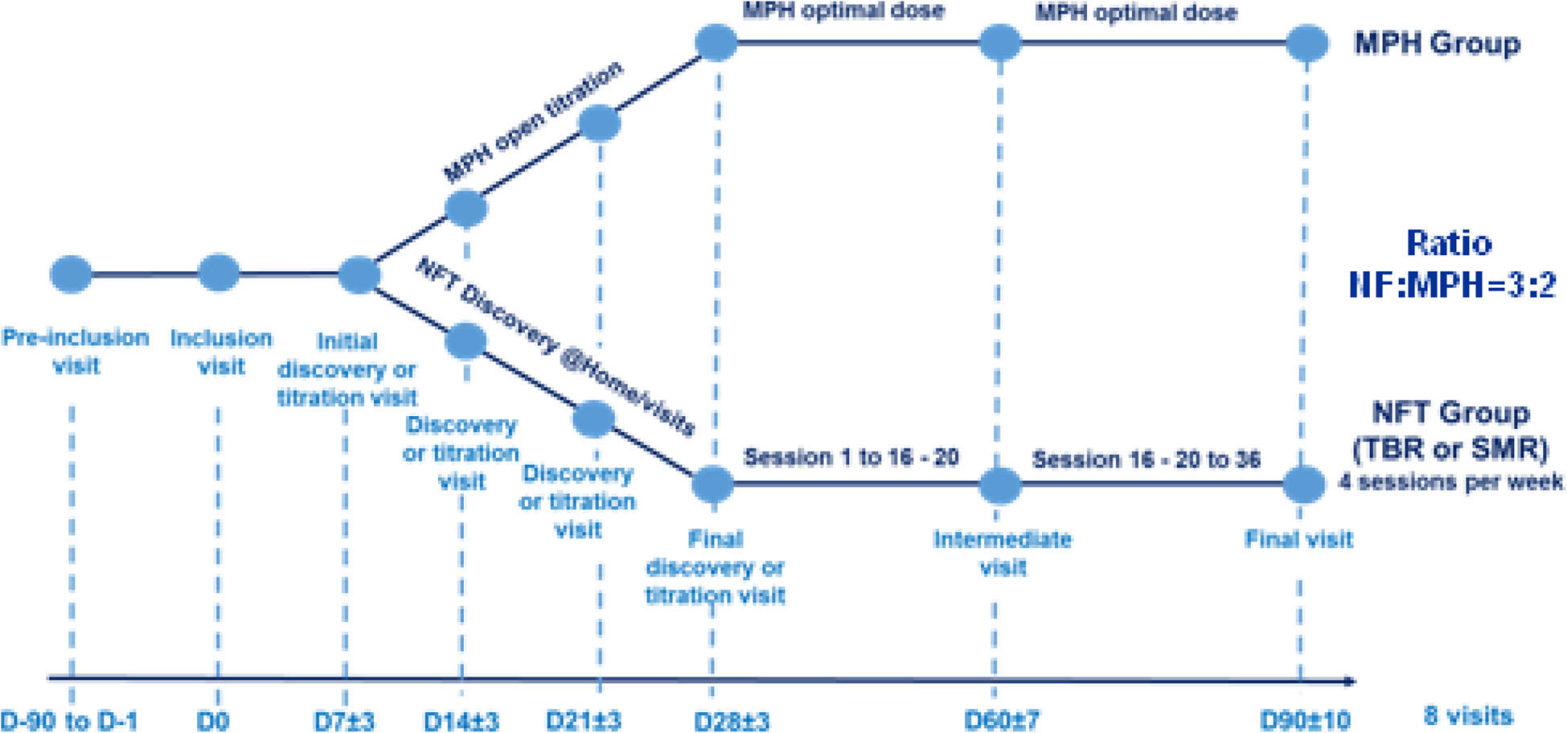
Flow chart study design

Figure 1 describes the design of the study and Table 1 shows the list of all assessments and their respective time of acquisition.

**Table 1:** Description of visits

Visit	Pre-inclusion	Inclusion	Discovery or open titration			Treatment period (MPH or NFB)		
			Initial discovery or titration	Discovery or titration	Discovery or titration	Final discovery or titration	Intermediate	Final
Day	D-90 to D-1	D0	D7 ± 3	D14 ± 3	D21 ± 3	D28 ± 3	D60 ± 7	D90 ± 10
Protocol explanation	X							
Inform consent form		X						
Inclusion/exclusion criteria		X						
Demographic data		X						
Medical/surgical history		X						
Medical examination		X						
qEEG		X					X	X
Randomisation		X						
MPH delivery			X	X	X	X	X	
NFB discovery session			X	X	X	X	X	
Kiddie-Sads	X							
ADHD RS parents		X					X	X
ADHD RS clinician		X					X	X
ADHD RS teacher		X						X
CGI-I score			X	X	X	X	X	X
CGI-S score		X	X	X	X	X	X	X
C-SSRS (Columbia suicide rating scale		X	X	X	X	X	X	X
SDSC		X	X	X	X	X	X	X
SDQ by teacher/parent		X						X
BRIEF		X						X
Conners 3 CPT		X						X
WASI 3 subtest (or WISC less 3 months)	X							
Concomitant treatments		X	X	X	X	X	X	X
Adverse event collection (spontaneous)			X	X	X	X	X	X
PAERS questionnaire		X	X	X	X	X	X	X

At pre-inclusion, children are screened and diagnosed to confirm their ADHD status. This evaluation includes the K-SADS interview [33], a semi-structured diagnostic interview designed to assess current and past episodes of psychopathology in children and adolescents according to DSM-IV criteria. At this visit cognitive function is tested with the WISC IV (Wechsler Intelligence Scale for Children) or with the WASI (Wechsler Abbreviated Scale of Intelligence; [34]) unless results from an IQ-test accomplished in the last 18 month are available.

Scales are filled out by one parent and it is requested that ratings are made by the same person throughout all assessment visits.

The description of the various visits is summarized in Table 1.

The inclusion visit includes the collection of demographic data, medical/surgical history, concomitant medication and a medical examination. Moreover, the following scales are completed: the ADHD-RS in its 3 versions (clinician, parents and teacher), the CGI-S, the C-SSRS, the SDSC, the SDQ (by parents and teachers) and the BRIEF. During the same visit a CPT and a qEEG are carried out (see Table 1).

qEEG is recorded with the same equipment used for neurofeedback training. The recording is performed under resting state conditions (while the subject is not performing any task) with one record of eyes-opened and another record of eyes-closed conditions (2-min each). At this visit a treatment group is assigned, according to the randomization list (NFT or MPH group).

Two distinct neurofeedback protocols are implemented in the application: the first is based on the down-training of the theta/beta ratio (noted “TBR”) in fronto-central areas, the second aims at enhancing the Sensori-Motor Rhythms (also called “SMR”) in central regions. The purpose of the first assessment is to determine the training protocol that will give the best result in the reduction of the ADHD symptoms. For that purpose, the theta/beta ratio is computed and compared to a threshold value equal to 4,5. Patients whose TBR is above 4,5 will follow a TBR down-training protocol whereas patients whose TBR is below 4.5 will follow a SMR up-training protocol. The decision made by the application has to be validated by the clinician. Both protocols give access to the same workflow in the application. Thus, children in the NFT group undergo either SMR or Theta/Beta training protocol according to their Theta Beta Ratio obtained during the qEEG.

During the four “discovery” (NF group) or titration visits (MPH group), CGI-I, and CGI-S, C-SSRS and SDSC are completed. Concomitant medications and adverse events are collected following the delivery of therapeutic units (MPH group) or training with the investigator (NF group).

During an intermediary visit the 3 versions of the ADHD-RS, CGI-I and CGI-S, C-SSRS and SDSC are completed and a qEEG is also done (see Table 1).

The final visit (three months after inclusion) includes the ADHD-RS in its 3 versions, the CGI-I and CGI-S score, the C-SSRS, the SDSC, the SDQ (by parents and teacher) and the BRIEF. This visit also comprises a CPT (CPT-3) and a qEEG (see Table 1).

### Interventions

#### Neurofeedback group

**Fig. 2:**
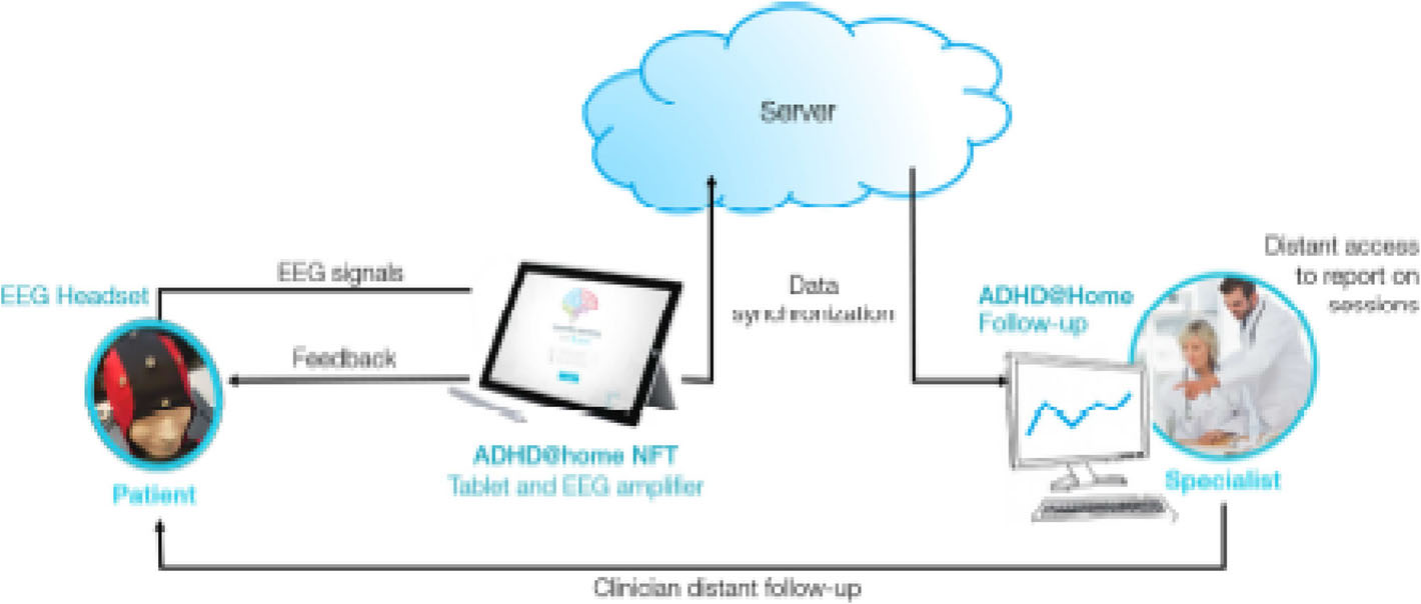
Overview of the ADHD@Home Device

The Mensia Koala™ medical device is composed of a software for personalized brain rehabilitation at home Training (“ADHD@Home NFT v1.1”) connected to an EEG amplifier and to an EEG cap. A baseline assessment starts every NF session; the data acquired on the tablet is automatically synchronized with a cloud server in order to make the personalized brain rehabilitation data retrievable on a secured web portal (“ADHD@Home Follow-Up v1.1”) (see Fig. 2). The software is used to record data from 8 AgCl scalp electrodes located at standard 10–20 locations (Fpz, Fz, F3, F4, Cz, C3, C4, and Pz) referenced and grounded to the left and right earlobes, respectively. The signals are amplified and digitalized over 24 bits 512 times per seconds. Pre-amplification analogue filters are designed to prevent aliasing so that frequency response of the acquisition is mostly flat between 0.01 and 100 Hz. Trained neuromarkers during NF are the following: SMR in C3 + Cz + C4 and TBR in F3 + Fz + F4 + Cz. The software is pre-installed on a tablet computer dedicated to the application. The tablet is battery powered and connected to a medical grade EEG signal amplifier, which is connected to the EEG cap by the mean of individual actively shielded cables protecting against common mode electromagnetic contaminations. All elements come in a transport case that patients take home. The application guides the user through his session featuring headset setup, calibration, and training protocol.

The EEG signal quality is controlled by a session-calibrated eye blink removal using Blind Source Separation [35] and a signal quality index (SQI) algorithm [36], both working in real-time to increase the training specificity by removing noise in the signal fed back to the user. The SQI is calibrated with data acquired in the clinic under expert supervision thereby setting the level of quality desired for sessions recorded in the home. The definition of typical EEG frequency bands is personalized to account for the discrepancies in the subjects’ brain maturation within our paediatric population. The individualized alpha peak frequency (iAPF) is defined as the maximum power value between 7 and 13 Hz at electrode Pz and used to define theta (iAPF− 5 to iAPF− 1 Hz), beta (iAPF+ 3 to iAPF-5 Hz), and SMR (iAPF+ 2 to iAPF+ 5 Hz). A qEEG assessment is first completed for all patients in order to calibrate the SQI, the iAPF, and determine the subject’s EEG profile. A theta/beta ratio (TBR) exceeding 4.5 is considered significant [37] (NCT02251743). During the initiation visit, the clinician presents the system and the neurofeedback application to the patient and his parents and supervises the first “discovery” neurofeedback training session.

The NF training session consists of five four-minute-long “active” NF blocs (with real-time feedback) and two two-and-a-half-minute-long “transfer” blocs (with only intermittent feedback). The total duration of a training session remains below 30-min. Two games sharing common design principles are implemented in the application and provided the subject with real-time rewards; the neuromarker activity is visualized as a moving bar and rewards are displayed through either a fishing of a puzzle game. The child is able to choose between two games at the beginning of each session. At the end of every block, a performance report is displayed to the user.

Three types of rewards are delivered:

- Classic reward: The classic reward increments the score on the session. The reward is obtained each time the neuromarker activity is above (up-training protocol) or below (down-training protocol) the threshold for a time longer than 500 ms (time-gating parameter). As long as the activity is maintained above/below the threshold, the reward is provided and the score incremented with the refreshing rate of the game.
- Cumulative booster: The cumulative booster commands a feedback element of the metaphor. A reward is given when the activity below (down-training protocol) or above (up-training protocol) the threshold reaches 3 s of cumulated time.
- Consecutive booster: The consecutive booster commands another element of the metaphor and acts as an extra reward. The reward is given when the activity is maintained below/above the threshold of 3 s.

Before starting the NF treatment, participants have neurofeedback discovery sessions. First these sessions occur at the clinical centre (1 to 4 discovery visits). Purpose of these meetings is to teach the child and parents how to use the system by themselves. The clinician shows the proceeding of a standard neurofeedback session and instructs the child to keep the moving bar above a certain threshold and to gain rewards (displayed either as fish ore treasures in the fishing game or as puzzle pieces in the puzzle game). When the participants are sufficiently autonomous to go home with the device and practice NF without the supervision of a clinician, they can begin the discovery sessions at home. Discovery sessions at home give the patient the opportunity to experience neurofeedback sessions alone or under his parents’ supervision. It occurs after one or several discovery visits. These sessions are not considered in the evaluation of the treatment efficacy. The number of discovery sessions performed at home is limited to a maximum of 10.

Participants receive an installation guide of the EEG cap and the ADHD@Home device. Each session begins with an impedance check to control the quality of contact of each electrode. The impedance is represented on the screen by a colored electrode’s map with different colors for good and bad contact quality.

The discovery phase ends with a “final” discovery session at the clinic. The first treatment phase starts when the patient is at ease with the system. Sixteeen to twenty neurofeedback sessions at home are scheduled from the specialist interface on a specific period lasting from a starting date, to the date of the next appointment at the clinic. During this phase, the patient logs in the application on average 4 times a week to complete neurofeedback sessions that have been prescribed to him. If he occasionally misses sessions, he can reschedule them on same week. Treatment resumes after the mid-assessment. The patient performs another 16 to 20 prescribed sessions at home, up to a total of 36 sessions, again with a frequency of 4 sessions per week. In total, 36 sessions (4 sessions per week for 9 weeks) should be done.

The specialist interface of the ADHD@Home solution allows the clinician to know if subjects have done their session. Clinicians have a remote follow up of patient performances and compliance via a web portal accessible on an Internet page. With this interface the specialist can follow the performance, the agenda of sessions and consult errors (connection, missing sessions,…) that have occurred during the training at home phase. Monitoring occurs every week. In the event that more than 2 or more sessions, out of 4, are missed during a given week, the clinician will call the parents of the child to enquire about the poor compliance. The goal is to understand the reason behind the missed sessions, to fix any issues that arise and to motivate the child and his parent to stick to the treatment regimen. They will be reminded that the missed sessions can be made up in subsequent weeks with a limit of 1 session per day.

ADHD@Home NF integrates algorithms for the identification of artefacts - non-EEG signals such as head movements, muscular contractions, blinks - in order to guarantee that personalized brain rehabilitation is performed based on clean brain signals. Artefacts interrupt the real time feedback of the software if they cannot be corrected sufficiently by the implemented artefact correction algorithms; this is displayed on the game by a cloud masking the sun or a changing background colour.

#### Methylphenidate group

There are two periods for this group: an open titration period and a treatment period. The open titration period lasts 3 weeks (from D3 to D28) and consists of 4 visits. The subject starts with 10 mg/day of extended-release methylphenidate/day. During the subsequent titration visits, the investigator decides if the dose is increased or not. The maximal dose is 60 mg/day. Titration is based on clinical experience of the investigator and the completed scales, taking into account both tolerance and risk/benefit ratio.

The treatment period lasts 2 months (from Day 28 to Day 90). During this period, the optimal dose is maintained.

### Assessments

#### Physical assessment

The physical examination includes a detailed clinical history, assessments of height and weight, blood pressure, and heart rate. Concomitant treatments are assessed in both treatment groups.

#### Psychometric assessments

##### ADHD rating scale IV (ADHD RS IV) [30, 38]

This scale is an 18-item scale with one item for each of the 18 symptoms contained in the DSM-IV diagnosis of ADHD: nine items make up the inattention subscale and the other nine the hyperactivity–impulsivity subscale. Each item is scored on a 0–3 scale. Clinician, parents and teachers will answer this scale to evaluate attention and hyperactivity/impulsivity.

The clinician version of ADHD Rating Scale, is a validated tool for assessing the severity of ADHD symptoms and change in symptom severity [38].

##### Behavior rating inventory executive function (BRIEF) [39]

This scale is a validated and standardized instrument that assesses executive functioning, including 8 subscales comprising 2 indices summed together in the Global Executive Composite.

##### Strengths and difficulties questionnaire (SDQ) [40]

The SDQ is a brief behavioral screening questionnaire assessing 25 attributes, some positive and others negative, which can be allocated to five scales (emotional symptoms, conduct problems, hyperactivity/ inattention, peer relationship problems, and prosocial behavior). These scales can be summed to calculate a total difficulties score with the advantage of being able to assess short-term changes [41].

##### Clinical global impression (CGI) scale [42]

The CGI is a clinician-administered tool providing a scoring of initial severity on the CGI–Severity Scale (CGI-S) from 1 to 7; subsequent improvements over time during treatment are rated using the improvement component (CGI-I). During follow-up visits, clinicians use the CGI-I 7-point scale to rate the patients’ total improvement based on comparison with their baseline assessment from 1 = very much improved to 7 = very much worse.

##### Pediatric adverse event rating scale (PAERS) [43]

The PAERS detects clinically important adverse events potentially but not necessary related to the drug. We will use the PAERS clinician interview form. The questionnaire includes 43 items to be asked in a patient-friendly language but recorded in the medical terminology. The administration time is 10 to 15 min. The PEARS is administered to both NF and MPH groups.

##### Columbia suicide severity rating scale (C-SSRS) [44]

The Columbia–Suicide Severity Rating Scale (C-SSRS) is designed to quantify the severity of suicidal ideation and behaviour. Four constructs are measured: The first is the severity of ideation (“severity subscale”), the second is the intensity of ideation subscale (“intensity subscale”), the third is the behavior subscale and the fourth is the lethality subscale, which assesses actual attempts. At inclusion, the « Lifetime/Recent version » will be used. At the other visits, the “Since the last visit version” will be used.

##### Sleep disturbance scale for children (SDSC) [45]

The Sleep Disturbance Scale for Children (SDSC) is a 26 item Likert-type rating scale which evaluates specific sleep disorders in children, and provides an overall measure of sleep disturbance suitable for use in clinical screening and research. Items are divided into six categories representing some of the most common sleep difficulties affecting adolescents and children: disorders of initiating and maintaining sleep, sleep breathing disorders, disorders of arousal/nightmares, sleep-wake transition disorders, disorders of excessive somnolence, and sleep hyperhidrosis (night-time sweating). The questionnaire is completed by a parent or caregiver on behalf of the child. Parents use a five-point, Likert-type scale. At the Final visit, the assessment will cover the period of the last month, rather than the six latest months.

##### Attention test

The Conners CPT 3 (Conners Continuous Performance Test 3) [32] is a validated and standardized computerized go/no-go and attention test measures attention and impulsivity. “Subjects have to react to target letters on the computer screen except to the letter X. The experiment comprises 6 blocs of 140 s each. Each block contains 54 targets (except the block 1: 53 targets) and 6 non-targets. The task lasts for 14 min. Participants observe computer generated letters presented at inter-stimulus intervals of 1, 2, and 4 s, with a display time of 250 ms. Results are described with different variables: correct hits (number of cases where a response occurs in presence of a target), commission errors (number of cases where a response occurs in presence of a non-target), mean reaction time (hit reaction time) and variability of hit reaction time (measured by standard deviation). These indicators are also recorded for every block and group. Commission errors are a measure of impulsivity”.

### Statistics

#### Sample-size calculation

The primary endpoint is the change from baseline (inclusion visit) to end of treatment (last visit) in the Clinician ADHD RS IV total score. The upper limit of the 90% confidence interval (1-sided) for the difference between the two groups in the primary endpoint is calculated. Non-inferiority is declared if the upper limit is less than a 4.5 points difference on the primary outcome. This non-inferiority margin comprised between half and 2/3 of the mean superiority threshold of 7.5 [46] and below the minimally clinically relevant difference of 6.6 [38] was considered a clinically acceptable.

With a non-inferiority bound estimated at 4.5 and a standard deviation estimated at 11.5 for the primary endpoint, and using a 3:2 ratio, the number of subjects required is 170. In anticipation of a 5% drop-out rate, the total number of patients required is 179. Therefore 179 patients, 72 in the MPH group and 107 in the NFT group will be recruited.

Statistical analysis is performed with SAS® version 9.4 or higher (SAS Institute North Carolina, USA).

### Analysis

#### Study populations

Different populations are defined:

- The total population: all patients enrolled in the study who signed the inform consent form;
- The modified Intent To Treat Population (mITT) / safety population: all randomized patients from total population who received at least one dose of methylphenidate for MPH group or who participated to the first neurofeedback session for NFT group;
- The Per Protocol (PP) population: patients from the ITT population with no major protocol deviations (primary population);

Demographic and baseline characteristics:

- Baseline characteristics will be described by treatment group on the mITT and on the PP at D90. Both groups will be compared as following:
  - For quantitative data, a Student t-test will be performed, or a non parametric Mann-Whitney test if the normality hypothesis is rejected.
  - For categorical data, a chi-square test will be used or a Fisher exact test depending on the sample size.

#### Primary endpoint analysis

The primary endpoint is the change from baseline (inclusion visit) to end of treatment (last visit) in the Clinician ADHD RS IV total score. The upper limit of the two-sided 90% confidence interval (one-sided 0.050 significance level) for the difference between the two groups in the primary endpoint [NFT: D90 - D0] - [MPH: D90 - D0] will be calculated. Non-inferiority will be declared if this upper bound is less than 4.5. Non-inferiority is declared if the upper limit is less than a 4.5 points difference on the primary outcome.

In case of non-homogeneity between treatment groups at baseline, a covariance analysis (SAS® Mixed procedure) will be performed on the difference D90-D0, with the baseline index as covariate and the treatment as factor. If necessary, other covariates could be tested. A sensitivity analysis including the center as covariate (SAS® Mixed procedure) will also be performed. The same analysis will be conducted with the investigator as covariate. This analysis will be performed on the PP population at D90. Results on the mITT population will be given for information purpose only. The only comparison provided for in the protocol is carried out on the main criterion. For all secondary efficacy endpoints analyses, statistical tests are given for information purpose only.

#### Secondary endpoint analysis

All secondary efficacy endpoints analyses will be performed on the PP population and statistical tests will be given for information purpose only. Analyses will use 2-sided tests at the 5% significance level, except the normality tested at the threshold of 1% (Shapiro-Wilk test).

The following analyses will be performed on patients for whom the studied endpoint is available at D0 and at least one post-baseline evaluation.

Clinician ADHD RS IV Total, Inattention and Hyperactivity Scores

- Parents ADHD RS IV Total, Inattention and Hyperactivity Scores
- Teacher ADHD RS IV Total, Inattention and Hyperactivity Scores
- Executive Function Tests by the Behavior Rating Inventory of Executive Function (BRIEF)
- The Conners Continuous Performance Test 3rd Edition (Conners CPT 3)
- In-school behavior by the Strengths and Difficulties Questionnaire (SDQ) scores
- Parents Strengths and Difficulties Questionnaire (SDQ) scores
- EEG signature evaluation including IAPF value, TBR value and SMR value.

Groups will be compared with:

- the chi-square test for categorical variables,
- the t-test for continuous variables (or the non-parametric Mann-Whitney test when the assumption of normality is questionable).

Statistical tests will be 2-sided with a 0.050 significance level.

Safety and adherence analysis:

The following numbers and percentages will be calculated and compared between the two groups: patients who experienced at least one adverse event (on the whole and by system/organ), at least one adverse event leading to discontinue the treatment, and at least one serious adverse event. All adverse events will be described in each group.

In addition, the use of concomitant medications will be summarized by therapeutic class using descriptive statistics. Concomitant medications will include all medications taken while the patient is treated with study drug.

The adherence will be described in each group during the treatment period:

- number of Neurofeedback Sessions completed for NFT patients,
- % of observance (number of taken pills / number of theoretical pills that should have been taken during the treatment period) of Methylphenidate for MPH patients.

Groups will be compared on the main baseline criteria, the clinician and parents ADHD-RS, the presence of at least one AE and presence of at least one ADE. Comparisons will follow the same methodology as described for the MPH/NFT comparisons.

Analyses of the NF subgroups:

In the NFT subgroup from the PP population, comparisons will be performed on the efficacy and safety data as following:

- Performers versus other NFT patients: a performer shows statistically significant changes in their EEG comparing resting state and training EEG averaged across 46 sessions;
- Learners versus other NFT patients: a learner shows statistically significant changes in their training EEG over 46 sessions;
- Fast Learners versus other NFT patients: a fast learner shows statistically significant changes in their training EEG over first 26 sessions only (mid-assessment);
- Transfer Learners versus other NFT patients: a transfer Learner shows statistically significant changes in their transfer training EEG over first 26 sessions.

These groups are defined using the longitudinal EEG data collected by the NEWROFEED RCT data and a listing of patients in each group will be provided by Mensia Technologies. The necessary details to replicate the identification of the exact same sub-populations can be obtained from the promoter.

Another comparison will be performed between Improvers versus non-improvers: an improver is a patient showing a reduction of the total clinician ADHD RS score of ≥25% from baseline.

#### Interim analysis

In order to monitor the EEG data quality, an interim analysis is planned on the first 50 patients; this will also permit us to compare the sessions at the clinic and at home. We have defined two criteria of data quality, which are known to affect EEG recordings:

- Electromagnetic (EM) contamination shows the contamination by main power;
- EMG contamination are generated by face and neck muscle.

We have also added two criteria that we believe are specific of our application:

- Slow frontal artefacts are characteristic of patients being given a too large EEG cap making the frontal electrode “float”;
- The “averaged” coefficients of the blink estimation are used to ensure that algorithms for blink correction were fed with clean data and converged appropriately.

## Discussion

This paper presents the protocol and design of a prospective, multicentre and randomized reference drug-controlled trial of NF, in children with ADHD. Innovative aspects of the NEWROFEED study are the personalisation of the NF training protocol according to the individual’s TBR ratio and the at-home training sessions. This is the first non-inferiority study between a personalized NF training device and pharmacological treatment.

NF is a non-pharmacological strategy that involves less complex cognitive strategies than cognitive remediation or psychotherapy despite clear behavioral and learning elements, involving physiological self regulation and state control.

In contrast to other types of brain signal (e.g. functional MRI), EEG provides a high temporal resolution offering quasi immediate feedback and a fine-grained analysis of learning mechanisms. Furthermore, EEG is less costly and more praticle than near-infrared spectroscopy (NIRS) and functional magnetic resonance imaging (fMRI). The EEG recording and its modifications could also constitute a physiological marker of ADHD and its evolution over the course of the treatment. The current trial will enable the assessment of non-inferiority of NF compared with standard treatment but will also evaluate the effects of NF training parameters (number and quality of sessions), and individual characteristics (learning speed and efficiency, transfer effects).

The presumed strengths of Mensia Koala™ are: artefact control, personalization, and home use all of which are targeted to improve clinical effectiveness. First, artefact control is implemented as a twofold strategy comprised of real time artefact correction (active shielding and eye blink removal [35] and detection (Riemannian SQI) [36]. Artefact control ensures the user is only rewarded based on his brain electrical activity and not on relatively high-amplitude unrelated artefacts (including muscle activity). It therefore prevents a subject to “cheat” by modifying (voluntarily or not) a physiological rather than neurophysiological variable. Then, the personalization in Mensia Koala is also intended to increase therapeutic efficacy by targeting unhealthy brain activity in a more specific fashion. The treatment is personalized at three levels: 1) The frequency bands for the training are personalized based on the individual Alpha Peak Frequency (iAPF) extracted from the patient brain activity, 2) Based on these personalized frequency bands, a neuromarker is automatically calculated by the software and compared to a reference value. 3) The adequate protocol is selected based on the computed neuromarker. First, iAPF bands are prefered because it has been reported that the correlation between the theta activity and the core symptoms of ADHD (ADHD-RS) was stronger when the frequency band definitions was corrected for iAPF [47]. This is particularly relevant for an ADHD pediatric population right within the age range affected by a pathophysiological brain maturation [48, 49]. Second, the NF protocol (SMR or TBR) is personalized in each child based on his EEG profile. The EEG phenotype is represented with the TBR because it was found to relate to clinical scales of ADHD as well as performance on executive function tests [50]. Despite failing at being used as a diagnostic neuromarker for ADHD [16, 51], it is a promising prognosis marker and has already reportedly being used as such (NCT02251743; [37]).

The fact that Mensia Koala can be used at home is also believed to contribute to its effectiveness mostly because the sessions occur in a more ecological environment, which could enhance the transfer of learned skills to real life situations. To maintain treatment quality in this context, data recorded at home is sent to a secured web portal offering remote monitoring of sessions compliance, quality, and performance (scores and neuromarkers). Several information are accessible through the interface in order to follow the patient progression during the treatment: alerts and errors logged by the application during the NF sessions at-home, a library with all sessions’ report generated during the study accessible for consultation or download, a statistics section with the evolution of neuromarkers and scores across sessions.

Other strengths of the NEWROFEED protocol are the sample size, the multi-centre study and the collaboration within the scientific committee of experts from the EEG domain and clinicians involved in ADHD.

The non-inferiority design versus MPH was chosen because, on the basis of previous studies we did not expect NF to be superior to the drug of reference and because the absence of superiority does not imply the treatments are equivalent. Advantages of choosing MPH as a comparator are its well documented effects across various settings and observers [52] and its availability (i.e. non-stimulant drugs are not available in all participating countries). It is also the first-line medication recommended in our target population [53]. However, the evidence of non-inferiority of NF against the robust effects of MPH does require technical innovation in NF delivery and remains a challenging issue.

A limitation of the current NEWROFEED protocol is that clinicians were not blind to group assignment. But due to the investigational products (pills versus NF with EEG during serious game on tablet), this will be a non-blinded study. It is difficult to assess children with ADHD in blind condition. One dimension of ADHD is impulsivity, it is difficult to conduct an interview without the children or his parents give some information about the treatment received (medication or neurofeedback).

Another possible limitation of the current NEWROFEED protocol is the absence of sham NF. Sham NF provides feedback unrelated to actual self-regulation performance. However, unsuccessful attempts to modulate neural activity may result in poor compliance and negative emotions incompatible with an optimal placebo condition. Apart from the ethical issues, the feasibility of a sham condition for neurofeedback is doubtful because it is likely to be detected [54] and because the first stage of the NFB learning process requires conscious control over the target variable [55]. We therefore compare NF to standard medical treatment with methylphenidate but include probably blinded assessments by teachers [6, 8, 10] and an objective attentional assessment by means of the CPT, a measurement sensitive to treatment effects [56]. Another limitation of our protocol is the lack of systematic follow up assessment given the relevance of long term effects expected for neurofeedback [57].

More generally, the implementation of NF also faces other challenges such as the necessity to provide a sufficient evidence-base for new treatments, appropriate training of clinicians, monitoring by accredited organisations and lastly, reimbursement possibilities for treatments meeting quality and efficacy criteria.

In conclusion, this trial is the first protocol and design of a prospective, multicentre and randomized reference drug-controlled trial in children with ADHD, using a personalized NF training device at home.

## Data Availability

Not applicable.

## Abbreviations

ADHD: Attention Deficit Hyperactivity Disorder
ADHD-RS: Attention Deficit Hyperactivity Disorder –rating scale
MPH group: Methylphenidate group
NF group: Neurofeedback Training Group
NF: Neurofeedback
qEEG: Quantitative EEG
SMR: Sensori-Motor Rhythms
TBR: Theta/beta ratio

## References

[1] Polanczyk G, de Lima MS, Horta BL, Biederman J, Rohde LA (2007). The worldwide prevalence of ADHD: a systematic review and metaregression analysis. Am J Psychiatry.

[2] Valmaggia LR, Latif L, Kempton MJ, Rus-Calafell M (2016). Virtual reality in the psychological treatment for mental health problems: an systematic review of recent evidence. Psychiatry Res.

[3] Loe IM, Feldman HM (2007). Academic and educational outcomes of children with ADHD. Ambul Pediatr.

[4] Taylor E, Dopfner M, Sergeant J, Asherson P, Banaschewski T, Buitelaar J (2004). European clinical guidelines for hyperkinetic disorder -- first upgrade. Eur Child Adolesc Psychiatry.

[5] Banaschewski T, Coghill D, Santosh P, Zuddas A, Asherson P, Buitelaar J (2006). Long-acting medications for the hyperkinetic disorders. A systematic review and European treatment guideline. Eur Child Adolesc Psychiatry.

[6] Sonuga-Barke EJ, Brandeis D, Cortese S, Daley D, Ferrin M, Holtmann M (2013). Nonpharmacological interventions for ADHD: systematic review and meta-analyses of randomized controlled trials of dietary and psychological treatments. Am J Psychiatry.

[7] Arns M, de Ridder S, Strehl U, Breteler M, Coenen A (2009). Efficacy of neurofeedback treatment in ADHD: the effects on inattention, impulsivity and hyperactivity: a meta-analysis. Clin EEG Neurosci.

[8] Cortese S, Brandeis D, Holtmann M, Sonuga-Barke EJ (2016). The European ADHD guidelines group replies. J Am Acad Child Adolesc Psychiatry.

[9] Hodgson K, Hutchinson AD, Denson L (2014). Nonpharmacological treatments for ADHD: a meta-analytic review. J Atten Disord.

[10] Micoulaud-Franchi JA, Geoffroy PA, Fond G, Lopez R, Bioulac S, Philip P (2014). EEG neurofeedback treatments in children with ADHD: an updated meta-analysis of randomized controlled trials. Front Hum Neurosci.

[11] Coben R, Evans J. Neurofeedback and neuromodulation techniques and applications. 1st ed. Academic Press; 2010. p. 450. Hardcover ISBN: 9780123822352. eBook ISBN: 9780123822369.

[12] Heinrich H, Gevensleben H, Strehl U (2007). Annotation: neurofeedback - train your brain to train behaviour. J Child Psychol Psychiatry.

[13] Bagdasaryan J, Quyen Mle V (2013). Experiencing your brain: neurofeedback as a new bridge between neuroscience and phenomenology. Front Hum Neurosci.

[14] Gevensleben H, Rothenberger A, Moll GH, Heinrich H (2012). Neurofeedback in children with ADHD: validation and challenges. Expert Rev Neurother.

[15] Arns M, Heinrich H, Strehl U (2014). Evaluation of neurofeedback in ADHD: the long and winding road. Biol Psychol.

[16] Barry RJ, Clarke AR, Johnstone SJ (2003). A review of electrophysiology in attention-deficit/hyperactivity disorder: I. qualitative and quantitative electroencephalography. Clin Neurophysiol.

[17] Liechti MD, Valko L, Muller UC, Dohnert M, Drechsler R, Steinhausen HC (2013). Diagnostic value of resting electroencephalogram in attention-deficit/hyperactivity disorder across the lifespan. Brain Topogr.

[18] Arns M, Conners CK, Kraemer HC (2013). A decade of EEG theta/Beta ratio research in ADHD: a meta-analysis. J Atten Disord.

[19] Lubar JF, Shouse MN (1976). EEG and behavioral changes in a hyperkinetic child concurrent with training of the sensorimotor rhythm (SMR): a preliminary report. Biofeedback Self Regul.

[20] Monastra VJ, Lynn S, Linden M, Lubar JF, Gruzelier J, LaVaque TJ (2005). Electroencephalographic biofeedback in the treatment of attention-deficit/hyperactivity disorder. Appl Psychophysiol Biofeedback.

[21] Doehnert M, Brandeis D, Straub M, Steinhausen HC, Drechsler R (2008). Slow cortical potential neurofeedback in attention deficit hyperactivity disorder: is there neurophysiological evidence for specific effects?. J Neural Transm (Vienna).

[22] Gevensleben H, Holl B, Albrecht B, Schlamp D, Kratz O, Studer P (2009). Distinct EEG effects related to neurofeedback training in children with ADHD: a randomized controlled trial. Int J Psychophysiol.

[23] Banaschewski T, Brandeis D (2007). Annotation: what electrical brain activity tells us about brain function that other techniques cannot tell us - a child psychiatric perspective. J Child Psychol Psychiatry.

[24] Thibault RT, Lifshitz M, Raz A (2016). The self-regulating brain and neurofeedback: experimental science and clinical promise. Cortex.

[25] Micoulaud-Franchi JA, Salvo F, Bioulac S, Fovet T (2016). Neurofeedback in attention-deficit/hyperactivity disorder: efficacy. J Am Acad Child Adolesc Psychiatry.

[26] Arns M, Strehl U (2013). Evidence for efficacy of neurofeedback in ADHD?. Am J Psychiatry.

[27] Riesco-Matías P, Yela-Bernabé JR, Crego A, Sánchez-Zaballos E. What do meta-analyses have to say about the efficacy of neurofeedback applied to children with ADHD? Review of previous meta-analyses and a new meta-analysis. J Atten Disord. 2019. 10.1177/1087054718821731. Advance online publication. 10.1177/1087054718821731 30646779

[28] Catala-Lopez F, Hutton B, Nunez-Beltran A, Page MJ, Ridao M, Macias Saint-Gerons D (2017). The pharmacological and non-pharmacological treatment of attention deficit hyperactivity disorder in children and adolescents: a systematic review with network meta-analyses of randomised trials. PLoS One.

[29] Van Doren J, Arns M, Heinrich H, Vollebregt MA, Strehl U, KL S (2019). Sustained effects of neurofeedback in ADHD: a systematic review and meta-analysis. Eur Child Adolesc Psychiatry.

[30] DuPaul GJ, Power TJ, Anastopoulos AD, Reid R (1998). ADHD rating-scale IV : checklist, norms and clinical interpretation.

[31] Steele M, Jensen PS, Quinn DM (2006). Remission versus response as the goal of therapy in ADHD: a new standard for the field?. Clin Ther.

[32] Conners CK (2014). Conners continuous performance test- third edition (Conners CPT 3) & Conners continuous auditory test of attention (Conners CATA): technical manual.

[33] Kaufman J, Birmaher B, Brent D, Rao U, Flynn C, Moreci P (1997). Schedule for affective disorders and schizophrenia for school-age children-present and lifetime version (K-SADS-PL): initial reliability and validity data. J Am Acad Child Adolesc Psychiatry.

[34] Axelrod BN (2002). Validity of the Wechsler abbreviated scale of intelligence and other very short forms of estimating intellectual functioning. Assessment.

[35] Barthelemy Q, Mayaud L, Renard Y, Kim D, Kang SW, Gunkelman J (2017). Online denoising of eye-blinks in electroencephalography. Neurophysiol Clin.

[36] Barthelemy Q, Mayaud L (2017). Scoring method based on improved signals analysis Mensia technologies.

[37] Kerson C (2013). A proposed multisite double-blind randomized clinical trial of neurofeedback for ADHD: need, rationale, and strategy. J Atten Disord.

[38] Zhang S, Faries DE, Vowles M, Michelson D (2005). ADHD rating scale IV: psychometric properties from a multinational study as a clinician-administered instrument. Int J Methods Psychiatr Res.

[39] Mahone EM, Cirino PT, Cutting LE, Cerrone PM, Hagelthorn KM, Hiemenz JR (2002). Validity of the behavior rating inventory of executive function in children with ADHD and/or Tourette syndrome. Arch Clin Neuropsychol.

[40] Goodman R (1997). The strengths and difficulties questionnaire: a research note. J Child Psychol Psychiatry.

[41] Holtmann M, Becker A, Banaschewski T, Rothenberger A, Roessner V (2011). Psychometric validity of the strengths and difficulties questionnaire-dysregulation profile. Psychopathology.

[42] Busner J, Targum SD (2007). The clinical global impressions scale: applying a research tool in clinical practice. Psychiatry (Edgmont).

[43] March J, Karayal O, Chrisman A (2007). CAPTN: the pediatric adverse event rating scale.

[44] Posner K, Brown GK, Stanley B, Brent DA, Yershova KV, Oquendo MA (2011). The Columbia-suicide severity rating scale: initial validity and internal consistency findings from three multisite studies with adolescents and adults. Am J Psychiatry.

[45] Bruni O, Ottaviano S, Guidetti V, Romoli M, Innocenzi M, Cortesi F (1996). The sleep disturbance scale for children (SDSC). Construction and validation of an instrument to evaluate sleep disturbances in childhood and adolescence. J Sleep Res.

[46] Tanaka Y, Rohde LA, Jin L, Feldman PD, Upadhyaya HP (2013). A meta-analysis of the consistency of atomoxetine treatment effects in pediatric patients with attention-deficit/hyperactivity disorder from 15 clinical trials across four geographic regions. J Child Adolesc Psychopharmacol.

[47] Vollebregt MA, van Dongen-Boomsma M, Slaats-Willemse D, Buitelaar JK, Oostenveld R (2015). How the individual alpha peak frequency helps unravel the neurophysiologic underpinnings of behavioral functioning in children with attention-deficit/hyperactivity disorder. Clin EEG Neurosci.

[48] Klimesch W (1999). EEG alpha and theta oscillations reflect cognitive and memory performance: a review and analysis. Brain Res Brain Res Rev.

[49] Doehnert M, Brandeis D, Imhof K, Drechsler R, Steinhausen HC (2010). Mapping attention-deficit/hyperactivity disorder from childhood to adolescence--no neurophysiologic evidence for a developmental lag of attention but some for inhibition. Biol Psychiatry.

[50] Clarke AR, Barry RJ, Dupuy FE, Heckel LD, McCarthy R, Selikowitz M (2011). Behavioural differences between EEG-defined subgroups of children with attention-deficit/hyperactivity disorder. Clin Neurophysiol.

[51] Gloss D, Varma JK, Pringsheim T, Nuwer MR (2016). Practice advisory: the utility of EEG theta/beta power ratio in ADHD diagnosis: report of the guideline development, dissemination, and implementation Subcommittee of the American Academy of neurology. Neurology.

[52] Cortese S, Adamo N, Del Giovane C, Mohr-Jensen C, Hayes AJ, Carucci S (2018). Comparative efficacy and tolerability of medications for attention-deficit hyperactivity disorder in children, adolescents, and adults: a systematic review and network meta-analysis. Lancet Psychiatry.

[53] National Institute for Health and Clinical Excellence (2018). Attention deficit hyperactivity disorder: diagnosis and management.

[54] Birbaumer N, Ramos Murguialday A, Weber C, Montoya P (2009). Neurofeedback and brain-computer interface clinical applications. Int Rev Neurobiol.

[55] Holtmann M, Pniewski B, Wachtlin D, Worz S, Strehl U (2014). Neurofeedback in children with attention-deficit/hyperactivity disorder (ADHD)--a controlled multicenter study of a non-pharmacological treatment approach. BMC Pediatr.

[56] Solanto MV, Wender EH, Bartell SS (1997). Effects of methylphenidate and behavioral contingencies on sustained attention in attention-deficit hyperactivity disorder: a test of the reward dysfunction hypothesis. J Child Adolesc Psychopharmacol.

[57] Van Doren J, Arns M, Heinrich H, Vollebregt MA, Strehl U, Loo SK (2019). Sustained effects of neurofeedback in ADHD: a systematic review and meta-analysis. Eur Child Adolesc Psychiatry.

